# 

**Published:** 1893-01-07

**Authors:** 


CONTENTS.

| | PAGE |
|---|---|
| **Autobiographical** | 227 |
| **Passing Topics** —The Other Side | 228 |
| **The Hospital Clinic**— | |
| St. Thomas's Hospital.—Hæmorrhoids | 235 |
| Bristol.—The Treatment of Tubercular Disease of the Joints | 236 |
| The Practitioners' Bookshelf.—"The Nationalisation of Health"—"A Manual of the Operations of Surgery for the Use of Senior Students, House Surgeons, and Junior Practitioners"—"Hygienic Measures in Infectious Disease"—"Inorganic Chemistry" | 237 |
| New Drugs, Appliances, and Things Medical.—Diabetic Foods—Messrs. Cadbury's Cocoas and Chocolates | 238 |
| **1892** | 229 |
| **Musical Amnesia** | 231 |
| **New Books and New Editions.**—Physiology made Easy | 232 |
| **Annotations.**—The Secret of Secrets—Winters Ancient and Modern—Medical Protection | 233 |
| **Notes and News** | 234 |
| **The Institutional Workshop**— | |
| Within the Hospitals.—The Royal Infirmary, Dundee | 239 |
| Practical Departments.—Cooking by Gas | 239 |
| Hospitals in India. I.—The Bengal Presidency—(1) Calcutta Medical Institutions | 240 |
| **Modern London.**—Faces in the Crowd | 241 |

**EXTRA SUPPLEMENT.—THE NURSING MIRROR.**

| | |
|---|---|
| **News from the Nursing World.**—A Friend's Picture—A New District Nurse—Princess Louise—President—A Popular Lecturer—A Lady Doctor as Lecturer—A Wise Memorial—Mill Road Infirmary—Practical Progress—Typhoid in Ireland—The Queen's Letter—The Late Miss Stains—One Warm Garment—Small Patients at Paddington—Rest and Fresh Air | cvii |
| **Christmas Festivities** | cx |
| **The Development of Children by Gymnastics. III.**—Dumb-bell Exercises | cix |
| **Our Christmas Parcels** | cxii |
| **Everybody's Opinion.**—Nursing in Typhoid | cxii |
| **Notes and Queries** | cxii |
| **Forty Years of Nursing.**—The Late Mrs. Wardroper and the Nightingale School | cxiii |

